# Stroke prevention of atrial fibrillation: Improving geographic under-use of contemporary antithrombotic approaches remains a challenge

**DOI:** 10.1016/j.ijcha.2021.100785

**Published:** 2021-04-23

**Authors:** L. Riesinger, R. Wakili, D. Dobrev

**Affiliations:** a Department of Cardiology and Vascular Medicine, West German Heart and Vascular Center Essen, University of Duisburg-Essen, Essen, Germany; b Institute of Pharmacology, West German Heart and Vascular Center, University Duisburg-Essen, Essen, Germany; c Montréal Heart Institute, University de Montréal, Montréal, Quebec, Canada; d Department of Molecular Physiology & Biophysics, Baylor College of Medicine, Houston, USA

Atrial fibrillation (AF) is the most common heart rhythm disorder worldwide. It could be highly symptomatic and is associated with increased morbidity and mortality [1]. Despite major progress in our understanding of AF pathophysiology the underlying mechanisms of AF are poorly understood [2]. AF is increasingly considered the result of an atrial cardiomyopathy caused by genetics, risk factors and comorbidities [3]. It is expected that better understanding of the AF-promoting atrial cardiomyopathy and its thromboembolic complications will ultimately lead to a better patient care, because current anti-arrhythmic approaches have moderate efficacy and substantial off-target effects. Oral anticoagulation (OAC) is one of the three main pillars of the ABC pathway for integrated AF management [4]. For many decades, OAC such as vitamin K antagonists (VKAs) have been the only available treatment option to prevent ischemic events in AF patients. However, given the narrow therapeutic range of VKAs with the concomitant need for regular INR monitoring and the patients’ as well as physicians’ concerns of bleeding complications, patients with AF have been often under-treated for many years, particularly in the Asian AF population [5]. The concerns about VKAs prompted the development of newer non-VKA oral anticoagulants (NOACs) with improved efficacy and a better safety profile as evidenced by large randomized controlled trials and real-world investigations [6], [7], [8], [9]. Additionally, NOACs might also exert antiarrhythmic effects [10]. Due to the easy handling of NOACs for both physicians and patients compared to VKAs, the introduction of NOACs a decade ago has progressively replaced the classical VKAs in the prevention of stroke in AF patients.

Despite intrinsic limitations of real world data, registries are an important source of information about geographic differences in AF treatment [11]. *The Global Registry on Long-Term Oral Antithrombotic Treatment in Patients With Atrial Fibrillation* (GLORIA-AF) was a global registry performed in different stages. Patients with newly diagnosed nonvalvular AF were enrolled into the registry during 3 separate phases. Phase 1 was conducted before approval of NOACs in each region. Phase 2 began in each country when dabigatran, the first approved NOAC, was introduced. Data including AF characteristics, pre-existing medical conditions and medications were collected. From November 2011 to December 2014, a total of 15,641 patients at 984 centers in 44 countries were enrolled in phase 2... Of those, about 70% were European or North American. About 20% of patients were enrolled in Asia, 6% in Latin America and 4% in the Africa/Middle East (AME) region. Interestingly, up to 20% in North America and up to 45% in Asia were either treated with acetylsalicylic acid or not at all, indicating lack of stroke prevention in a substantial proportion of patients. The underuse of anticoagulants might be a consequence of concordance with the U.S. guidelines at that time, which advised antiplatelet therapy or no antithrombotic therapy as an alternative to OAC in patients with CHA2DS2-VASc scores of 1 [12]. Of note, the 4% of patients enrolled in the AME region had a much higher anticoagulation rate (only 10% received ASA and <2% remained untreated), with patients in Europe having the highest anticoagulation rate [13].

In this issue of the International Journal of Cardiology Heart & Vasculature Azar et al. report about the treatment of patients from the Africa/Middle-East Region Sub-Study of the GLORIA-AF [14]. They compare the baseline characteristics, concomitant medication, medical treatment as well as effectiveness and safety outcomes in this particular patient collective to the overall data of the GLOARIA-AF registry, identifying a slightly younger patient collective with more comorbidities, but comparable CHA_2_DS_2_-VASc- and HAS-BLED-scores. Most of the patients enrolled in the AME region were treated with a reduced dabigatran dose (110 mg twice daily), in contrast to the global cohort, where the 150 mg dose twice daily was the most frequent option. Of note, ischemic events and bleeding complications were very rare in this cohort, perhaps because of using a lower dose of dabigatran. In a comprehensive overview, the authors relate their real-world data to the outcome of the randomized “RE-LY” trial, and to the only multinational prospective registry on AF and OAC performed in this region, the Gulf SAFE registry that was performed in “before-NOAC-times”, providing complementary evidence to those from randomized controlled trials, particularly because the latter usually under-represent a particular region or ethnicity. A direct comparison to the randomized RE-LY trial (that included 18,113 patients, compared to only 600 in this registry) cannot be made, as most of the patients enrolled in the AME region were treated with a reduced dose of 110 mg dabigatran twice daily. Moreover, the investigated patients were younger (67 vs 71 years in the RE-LY trial), with a higher proportion of comorbidities like arterial hypertension and diabetes, but less patients with a history of stroke [6]. Although a direct comparison cannot be made and the event rate in the reported registry was very low, dabigatran use was associated with a higher efficacy and a better safety [14]. The Gulf SAFE registry enrolled 2043 consecutive patients with AF from Kuwait, Bahrain, Qatar, United Arab Emirates, Oman and Yemen which were younger (57 vs. 67 years) and had fewer comorbidities, as indicated by the lower CHA_2_DS_2_-VASc score. Still, stroke/transient ischemic attack rates (4.2%) and all-cause mortality (13%) were much higher in this registry (compared to less than 1%, or less than 2% respectively, in the GLORIA-AF sub study [14] presumably due to insufficient use of anticoagulation, because in the Gulf SAFE registry only 58% of patients received a VKA and of those only 50% underwent regular INR checks [15]. This highlights that use of NOACs instead of VKAs is associated with a better treatment of AF patients independent of ethnicity or geographic region.

In conclusion, prospective observational registries of AF patients from different world regions, as exemplified by Azar et al. [14], provide important insights into region-specific patterns of AF management, healthcare system features and unmet needs along with knowledge gaps, thereby helping to improve the management of AF patients and, ultimately, their outcome. Prospective randomized clinical trials are clearly required to determine the efficacy and safety of OACs in the prevention of thromboembolic events in the many in randomized clinical trials under-represented regions and ethnicities of the world.

## Funding sources

This study is supported by grants from National Institutes of Health [R01HL136389, R01HL131517 and R01HL089598 to D.D.], the German Research Foundation [DFG, Do 769/4-1 to D.D.], and the European Union (large-scale integrative project MEASTRIA, No. 965,286 to D.D.).

## Conflict of interest

L. Riesinger has no conflicts of interest.

R. Wakili obtained research grants from DZHK (Deutsches Zentrum für Herz-Kreislaufforschung) and Boston Scientific and personal fees from Bayer, Boehringer, Daichi Sankyo, Pfizer, Bristol-Myers-Squibb, Boston Scientific, and Biotronik outside the submitted work.

D. Dobrev is member of the Scientific Advisory Boards of Omeicos Therapeutics GmbH and Acesion Pharma.
